# Drug-Coated Balloon Angioplasty for Isolated Medina 0,0,1 Ostial Side-Branch Lesions: Procedural Outcomes, Bailout Stenting, and 12-Month Clinical Follow-Up

**DOI:** 10.3390/medicina62091620

**Published:** 2026-08-22

**Authors:** Ebru Şahin, Cansu Akdeniz, Begüm Sayın, Hakan Aksoy, Fehmi Kaçmaz, Ali Oto

**Affiliations:** Department of Cardiology, Memorial Ankara Hospital, 06520 Çankaya, Ankara, Türkiye; scansuakdeniz@gmail.com (C.A.); begumyts@yahoo.com (B.S.); haksoy63@yahoo.com (H.A.); kacmazfehmi@gmail.com (F.K.); alioto@tksv.org (A.O.)

**Keywords:** drug-coated balloon angioplasty, Medina 0,0,1, ostial side-branch lesion, coronary bifurcation, lesion preparation, bailout stenting, clinical outcomes

## Abstract

*Background and Objectives:* Stenting isolated Medina 0,0,1 ostial side-branch lesions can result in geographic miss or protrusion into the main vessel. Drug-coated balloon (DCB) angioplasty avoids a permanent implant, but lesion-specific outcome data remain limited. We assessed procedural performance, bailout stenting, and 6- and 12-month outcomes after an intended DCB-only strategy. *Materials and Methods:* In this retrospective, single-center observational cohort study, we screened 170 patients who underwent coronary DCB treatment at a single center between 1 January 2023 and 31 July 2025. Thirty-one patients with de novo isolated Medina 0,0,1 lesions were included. The primary endpoint was 12-month target lesion failure (TLF), defined as cardiovascular death, target-vessel myocardial infarction, or clinically driven target lesion revascularization (TLR). *Results:* DCB-only strategy success was achieved in 29/31 patients (93.5%), post-DCB angiographic success in 29/31 (93.5%), and study-defined overall procedural success in 31/31 (100%). Final TIMI grade 3 flow after DCB was present in 30/31 patients; no flow-limiting dissection occurred. Two patients required bailout stenting, one because of deterioration to TIMI grade 2 flow and one because of residual stenosis >30% with recoil. Thirty patients were alive with documented follow-up at 12 months; one patient died from a non-cardiovascular cause at month 1. No TLF was recorded within 12 months. One clinically driven TLR for target-lesion restenosis occurred at month 20, outside the prespecified window. *Conclusions:* In this selected cohort, an intended DCB-only strategy avoided stent implantation in most patients and had a low bailout rate. The findings support procedural feasibility but do not establish comparative efficacy. Larger prospective studies are required.

## 1. Introduction

Coronary bifurcation lesions account for about 15–20% of percutaneous coronary interventions, and isolated Medina 0,0,1 lesions represent only 3–5% of bifurcation procedures [1,2]. Their rarity does not make them simple to treat. The side branch is often small, the ostium may be fibrotic or calcified, and recoil may follow balloon dilatation. Stent placement is difficult because incomplete ostial coverage leaves geographic miss, whereas excessive coverage extends the stent into a main vessel without relevant disease. Contemporary registries report clinically relevant rates of repeat revascularization and other adverse cardiovascular events after PCI [3,4].

Drug-coated balloon angioplasty delivers an antiproliferative drug without leaving a permanent metallic scaffold or polymer. For an isolated side-branch ostial lesion, it can preserve main-vessel anatomy and future access to both branches [5,6]. The trade-off is the absence of mechanical support. The Drug-Coated Balloon Academic Research Consortium defines an acceptable result before DCB treatment as residual stenosis ≤30% by visual estimation, TIMI grade 3 flow, and no flow-limiting dissection [5]. Recoil, impaired flow, or a major dissection still requires stent implantation.

Evidence for DCB-only treatment of Medina 0,0,1 lesions remains limited to small prospective or observational cohorts. Vaquerizo et al. reported the first dedicated prospective series, and Kang et al. later compared DCB- and drug-eluting stent–based treatment in an observational analysis [7,8]. No randomized trial has focused specifically on this lesion subtype. We examined the feasibility of an intended DCB-only strategy, the circumstances that led to bailout stenting, and procedural and 6- and 12-month clinical outcomes.

## 2. Methods

### 2.1. Study Design and Patient Population

This retrospective, single-center observational cohort study was conducted at Memorial Ankara Hospital, Ankara, Türkiye. The catheterization laboratory database was reviewed for patients who underwent coronary angiography and DCB treatment between 1 January 2023 and 31 July 2025. Of 170 DCB-treated patients screened, 48 had a side-branch lesion, and 33 met the angiographic criteria for a de novo isolated ostial side-branch Medina 0,0,1 lesion. Two patients without follow-up data were excluded, leaving 31 patients in the study cohort. The sample comprised all patients who met the prespecified eligibility criteria during the study period; no formal sample-size calculation was performed.

Eligible patients were aged ≥18 years, underwent DCB angioplasty of the target side-branch ostium, and had adequate procedural and follow-up records. Chronic and acute coronary syndrome presentations were included. Exclusion criteria were in-stent restenosis, bifurcation anatomy other than Medina 0,0,1, significant disease in the adjacent main-vessel segments, absence of DCB treatment, or insufficient records.

The retrospective study protocol and the secondary use of routinely collected clinical data were reviewed and approved by the Ankara Memorial Hospital Ethics Committee (Decision No. 202607-02-02; 22 July 2026). The committee waived the requirement for individual informed consent because the study used only existing electronic health records, coronary angiography and procedural reports, laboratory data, and routine follow-up records and involved no additional examinations, interventions, surveillance angiography, or patient contact. Data were coded or de-identified before analysis, and no direct identifiers were included in the analytic dataset. The study complied with the Declaration of Helsinki. This report was prepared in accordance with the Strengthening the Reporting of Observational Studies in Epidemiology (STROBE) statement for cohort studies. Treatment decisions were made during routine clinical care before and independently of the present retrospective analysis; no participant was prospectively assigned to an intervention for research purposes, and clinical trial registration was therefore not applicable.

Baseline comorbidities, clinical presentation, and left ventricular ejection fraction were abstracted from the hospital electronic record; angiographic and procedural variables were obtained from the catheterization laboratory database, procedure reports, and review of the index angiograms. Clinical outcomes were identified from hospital electronic records and documented outpatient assessments. Baseline diagnoses and clinical events were recorded as documented in the source records, and the same prespecified eligibility criteria were applied throughout the screening period.

For this retrospective analysis, clinical presentation at the index admission was classified from contemporaneous routine-care records. Unstable angina was defined as angina at rest or with minimal exertion, generally lasting >20 min; new-onset severe angina; or a crescendo pattern characterized by increasing frequency, duration, or severity or a lower exertional threshold, in the absence of acute cardiomyocyte injury. Patients meeting these clinical features underwent high-sensitivity cardiac troponin (hs-cTn) measurement at presentation and at 1 h as part of routine care. Classification as unstable angina required both hs-cTn values to remain below the assay-specific 99th percentile without a rise or fall consistent with myocardial infarction. hs-cTn was not measured systematically in the entire DCB cohort, but was obtained when the presentation raised suspicion of unstable angina or another acute coronary syndrome. All patients underwent 12-lead electrocardiography and transthoracic echocardiography as part of routine clinical assessment [9].

### 2.2. Lesion Assessment and DCB Procedure

The indication for angiography and intervention was based on clinical presentation. Lesions were classified using the Medina system on the index angiogram. The treated side branch, visually estimated diameter stenosis, reference vessel diameter, lesion length, and baseline TIMI flow were recorded. Formal quantitative coronary angiography was not performed; all reported angiographic measurements were therefore based on visual estimation.

During each index procedure, the lesion and its angiographic response to lesion preparation were evaluated in at least two angiographic projections by two interventional cardiologists, and procedural decisions were made by consensus. For the present retrospective analysis, stored pre-intervention angiograms, including non-contrast fluoroscopic images obtained before contrast injection and contrast-enhanced cineangiograms, and procedural records were reviewed. Coronary calcification was classified retrospectively according to the European Association of Percutaneous Cardiovascular Interventions (EAPCI) angiographic criteria [10]. Moderate calcification was defined as radiopaque density visible during cardiac motion and involving one side of the vessel wall, whereas severe calcification was defined as radiopacity visible without cardiac motion before contrast injection and generally involving both sides of the arterial wall. Angiographically visible calcification that did not meet these criteria was classified as mild. Because intravascular imaging was not available, calcium arc, thickness, and depth could not be quantified.

All lesions were prepared before DCB delivery using either a semi-compliant or non-compliant balloon. During the index procedure, balloon type was selected according to angiographic lesion characteristics, including the visually assessed calcific burden, and the operators’ joint judgment. Non-compliant balloons were selected for lesions with relatively more pronounced angiographic calcification when greater dilatation force was considered necessary. Predilatation balloon diameter was generally selected to achieve an approximate balloon-to-reference vessel diameter ratio of 0.8–1.0 based on visual angiographic assessment. In very tight lesions, initial preparation could begin with a smaller nominal balloon to facilitate lesion crossing; adequacy was determined from the post-predilatation angiographic result before DCB treatment. For the retrospective analysis, lesion preparation was considered adequate when the post-predilatation angiogram showed residual stenosis ≤30%, TIMI grade 3 flow, and no flow-limiting dissection. Procedural records showed that DCB angioplasty was performed only after these conditions had been met. A DCB with a diameter corresponding to an approximate balloon-to-reference vessel ratio of 0.8–1.0 and sufficient length to extend 2–3 mm beyond both the proximal and distal margins of the predilated segment was then selected and inflated at nominal pressure for approximately 90 s. Balloon dimensions and inflation pressure, together with DCB dimensions and inflation duration, were obtained from procedural records. The platforms used were MagicTouch (Envision Scientific Pvt. Ltd., Surat, India), a sirolimus-coated balloon, and Prevail (Medtronic, Minneapolis, MN, USA), a paclitaxel-coated balloon. Device selection was at the operator’s discretion. After DCB treatment, final angiography was obtained to assess residual stenosis, final TIMI flow, dissection, recoil, the need for bailout stenting, and overall procedural success. Dissections were classified angiographically as type A, B, or C and additionally categorized as flow-limiting or non-flow-limiting.

DAPT duration was determined by clinical presentation: the 14 patients without an acute coronary syndrome received 6 months, whereas the 17 patients with an acute coronary syndrome received 12 months. Both patients who underwent bailout stenting belonged to the chronic coronary syndrome group and received DAPT for 6 months; bailout stenting did not independently prolong treatment. Continued use was documented during follow-up, and no unplanned interruption was recorded.

### 2.3. Procedural Definitions

DCB-only strategy success was defined as completion of the intended treatment with post-DCB angiographic success and without bailout stenting. Post-DCB angiographic success by visual estimation required residual stenosis ≤30%, final TIMI grade 3 flow, and no flow-limiting dissection or acute target-vessel occlusion.

Study-defined overall procedural success required an acceptable final angiographic result after DCB-only treatment or bailout stenting, without in-hospital cardiovascular death, urgent target lesion revascularization, or a major clinically apparent complication. Bailout stenting therefore constituted DCB-only strategy failure but did not preclude overall procedural success when an acceptable final PCI result was achieved.

Bailout stenting was defined as stent implantation after DCB treatment for TIMI flow ≤2 or deteriorating coronary flow, flow-limiting dissection, acute occlusion, excessive recoil, or a persistent unacceptable angiographic result. Cardiac biomarkers were not routinely measured after elective procedures; periprocedural myocardial infarction was therefore recorded only when clinically diagnosed rather than through systematic biomarker surveillance.

### 2.4. Study Endpoints

The primary endpoint was target lesion failure at 12 months, defined as cardiovascular death, target-vessel myocardial infarction, or clinically driven target lesion revascularization. Secondary endpoints were DCB-only strategy success, post-DCB angiographic success, overall procedural success, final TIMI grade 3 flow, flow-limiting dissection, bailout stenting and its indication, and 6-month target lesion failure. The individual components of TLF were assessed at 6 and 12 months. Other outcomes included target-vessel revascularization, any myocardial infarction, all-cause and non-cardiovascular death, and target lesion thrombosis or occlusion. Non-cardiovascular death was reported separately and was not included in target lesion failure.

### 2.5. Clinical Follow-Up

Follow-up information was obtained from hospital electronic records and documented outpatient assessments. Clinical outcomes were evaluated at predefined 6- and 12-month time points. Clinical follow-up and electronic-record review were administratively closed on 22 July 2026. No surviving participant in the analyzed cohort was lost before the 6- or 12-month landmark. Events after 12 months were excluded from the primary endpoint and summarized descriptively as extended follow-up. Because follow-up beyond 12 months was not uniform, its duration and completeness at clinically relevant landmarks were summarized in Supplementary Table S1. Overall observation time was summarized using the median, interquartile range, and range.

### 2.6. Statistical Analysis

Analyses included all 31 patients in whom the DCB strategy was attempted. Continuous variables were summarized as mean and standard deviation when approximately normally distributed and as median with interquartile range otherwise. Categorical variables were reported as *n*/N (%), with the available denominator shown when data were missing. Immediate procedural proportions are presented with two-sided exact 95% confidence intervals calculated using the Clopper–Pearson method. Clinical events were counted within the prespecified 6- and 12-month windows. Because one patient died from a non-cardiovascular cause before both landmarks, clinical outcomes are reported as event counts together with follow-up completeness rather than as simple binomial proportions. Events after 12 months were summarized separately. No missing-data imputation was performed. The small sample, absence of a control group, and limited event count precluded multivariable modeling, predictor analysis, subgroup comparison, or comparative hypothesis testing. No sensitivity analyses were performed. Analyses were conducted using Python 3.12.13.

## 3. Results

### 3.1. Patient Selection and Follow-Up Completeness

**Figure 1:**
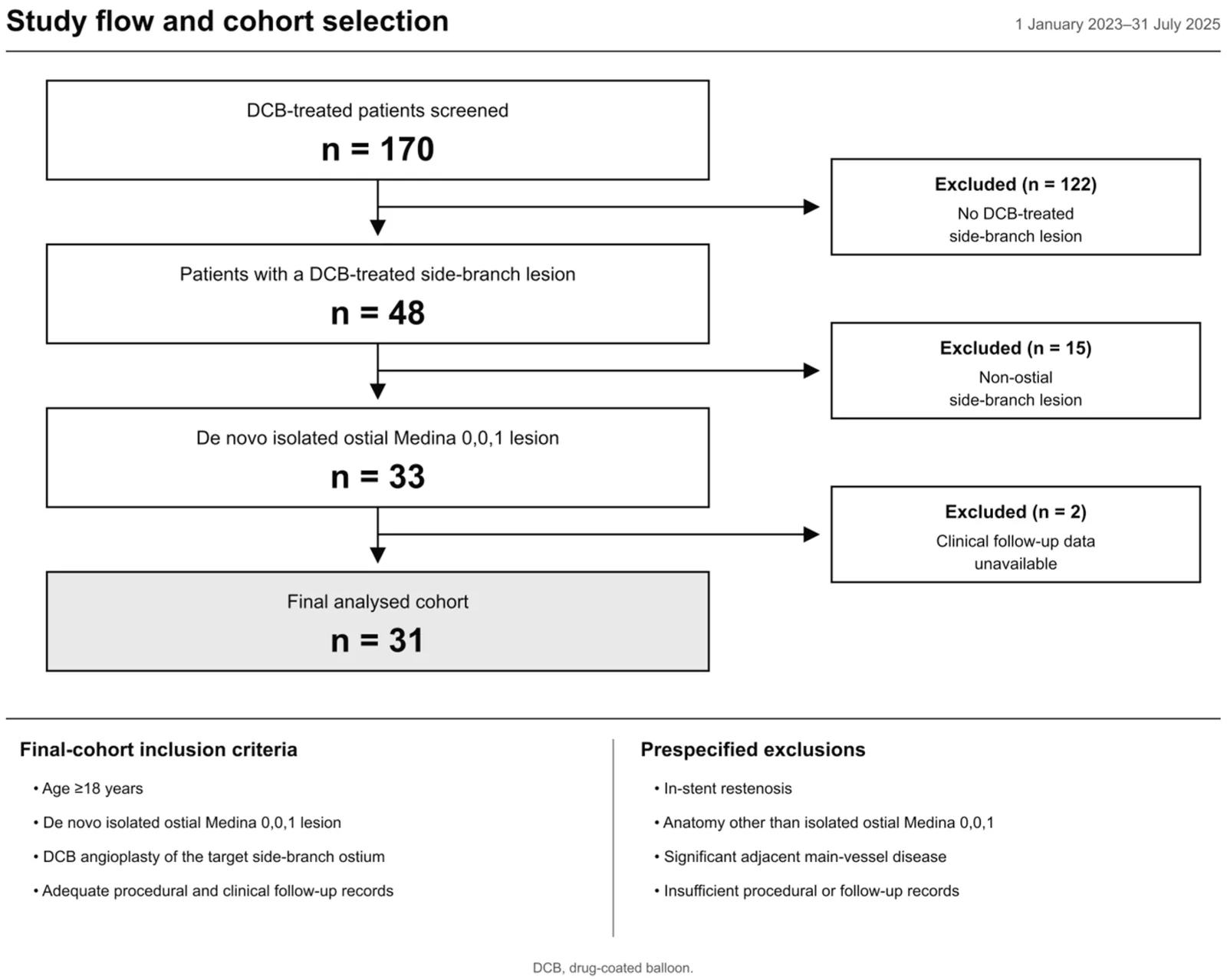
Study flow and cohort selection. Of 170 DCB-treated patients screened, 122 without a DCB-treated side-branch lesion were excluded. Among the remaining 48 patients, 15 were excluded because the side-branch lesion was non-ostial, and two were excluded because clinical follow-up data were unavailable. The final analyzed cohort comprised 31 patients with de novo isolated ostial Medina 0,0,1 lesions. DCB, drug-coated balloon.

The final cohort comprised 31 patients treated between 7 March 2024 and 22 July 2025. Median follow-up was 13 months (IQR, 12–17; range, 1–28). Thirty patients were alive and had documented clinical follow-up at both 6 and 12 months. One patient died from a non-cardiovascular cause after 1 month. Beyond the 12-month landmark, 19 patients had more than 12 months of record-based follow-up; 7 had at least 18 months, 4 had at least 20 months, and 3 had at least 24 months of follow-up. The duration and completeness of extended follow-up are summarized in Supplementary Table S1. The study selection process is summarized in Figure 1.

### 3.2. Baseline Clinical Characteristics

Median age was 65 years (IQR, 58–71), and 5 patients (16.1%) were women. Diabetes mellitus was present in 16 (51.6%), hypertension in 22 (71.0%), hyperlipidemia in 25 (80.6%), and a history of smoking in 19 (61.3%). Sixteen patients (51.6%) had undergone previous percutaneous coronary intervention, and 5 (16.1%) had a prior myocardial infarction. Clinical presentation was stable in 13 patients (41.9%), unstable angina in 13 (41.9%), non-ST-segment elevation myocardial infarction in 2 (6.5%), and ST-segment elevation myocardial infarction in 2 (6.5%); one patient (3.2%) was asymptomatic. Overall, 17 patients (54.8%) presented with an acute coronary syndrome. Median left ventricular ejection fraction was 56.5% (IQR, 55–60; *n* = 30).

### 3.3. Angiographic and Lesion Characteristics

The target coronary territory was the left anterior descending artery in 18 patients (58.1%), the circumflex artery in 11 (35.5%), and the right coronary artery in 2 (6.5%). Median reference vessel diameter was 2.5 mm (IQR, 2.25–2.5; *n* = 29), median lesion length was 18 mm (IQR, 15–20; *n* = 28), and median baseline stenosis was 80% (IQR, 80–90). Baseline TIMI flow was grade 3 in 28 patients (90.3%), grade 2 in 2 (6.5%), and grade 0 in 1 (3.2%). Calcification was recorded in 11 patients (35.5%) and a thrombotic lesion in 2 (6.5%).

### 3.4. Procedural Characteristics and Immediate Outcomes

A sirolimus-coated balloon was used in 28 patients (90.3%) and a paclitaxel-coated balloon in 3 (9.7%). Median first DCB diameter was 2.5 mm (IQR, 2.25–2.5), and median first DCB length was 20 mm (IQR, 15–20). Three patients (9.7%) required a second DCB to complete longitudinal coverage of the predilated segment. In these cases, the first DCB did not fully encompass the prepared segment together with the intended proximal and distal margins; an overlapping second DCB was therefore inflated. Second DCB use was not prompted by dissection, recoil, impaired coronary flow, or device failure. Angiographically visible calcification was present in 11/31 lesions (35.5%); all were classified as mild, and none met the EAPCI criteria for moderate or severe angiographic calcification. Among the calcified lesions, 7 were prepared using a semi-compliant balloon and 4 using a non-compliant balloon. In the overall cohort, semi-compliant balloons were used in 27/31 patients (87.1%) and non-compliant balloons in 4/31 (12.9%). On retrospective review, all lesions met the study-defined angiographic criteria for adequate lesion preparation before DCB treatment. No scoring or cutting balloon, atherectomy, or intravascular lithotripsy was used. Median residual stenosis after lesion preparation was 10% (IQR, 0–20). Baseline, angiographic, and procedural characteristics are summarized in Table 1.

**Figure 2:**
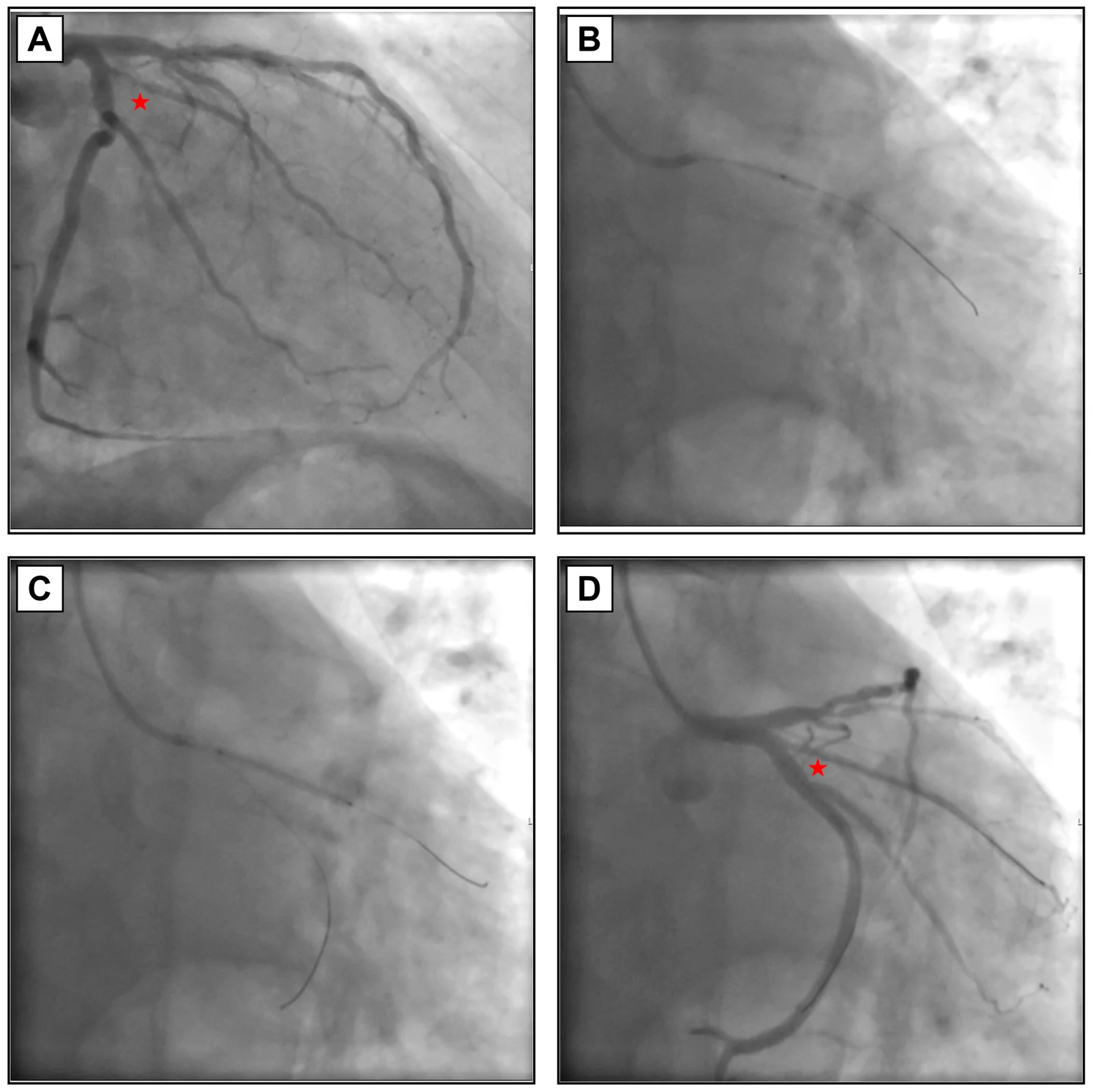
Representative angiographic sequence of DCB angioplasty for an isolated ostial Medina 0,0,1 side-branch lesion. (**A**) Severe OM1 ostial stenosis with an estimated lesion length of 19 mm and reference vessel diameter of 2.0 mm (red star). (**B**) Because of the tight ostial stenosis, lesion preparation was initiated with a nominal 1.5 × 15 mm Artimes (BrosMed Medical Co., Ltd., Dongguan, China) semi-compliant balloon inflated at 14 atm (nominal balloon-to-vessel ratio, 0.75; expected compliance-chart diameter, approximately 1.7 mm), followed by a 3–4 mm distal overlapping inflation. Post-predilatation angiography demonstrated approximately 10% residual stenosis, TIMI grade 3 flow, and no flow-limiting dissection. (**C**) A 2.0 × 25 mm MagicTouch sirolimus-coated balloon was inflated at 8–10 atm for 90 s. (**D**) Final angiography demonstrated TIMI grade 3 flow, residual stenosis ≤30% by visual estimation, and no flow-limiting dissection (red star). The compliance-chart diameter represents an expected in vitro value rather than a measured in-lesion diameter. DCB, drug-coated balloon; OM1, first obtuse marginal branch; TIMI, Thrombolysis in Myocardial Infarction.

TIMI grade 3 flow was present after lesion preparation in all patients and after DCB treatment in 30/31 (96.8%; 95% CI, 83.3–99.9%). Two patients (6.5%) had type A dissection; no type B or type C-or-greater dissection and no flow-limiting dissection occurred. Post-DCB angiographic success by visual estimation was achieved in 29/31 (93.5%), DCB-only strategy success in 29/31 (93.5%; 95% CI, 78.6–99.2%), and study-defined overall procedural success in 31/31 (100%). There was no emergency coronary artery bypass grafting (CABG) or in-hospital death. Immediate procedural and clinical outcomes are summarized in Table 2. A representative procedural sequence is shown in Figure 2.

### 3.5. Bailout Stenting

Bailout stenting was required in 2/31 patients (6.5%; 95% CI, 0.8–21.4%). Both cases involved angiographically non-calcified diagonal-branch lesions with a visually estimated reference vessel diameter of 2.5 mm. The first lesion was 10 mm long and exhibited 70% diameter stenosis. Predilatation with a 2.5 × 10 mm semi-compliant balloon reduced residual stenosis to approximately 10%, after which a 2.5 × 15 mm MagicTouch sirolimus-coated balloon was inflated for 90 s. Following DCB treatment, coronary flow deteriorated from TIMI grade 3 to grade 2. An intracoronary vasodilator was administered, followed by an observation period of approximately 5 min. Repeat angiography in two distinct projections confirmed persistent TIMI grade 2 flow. Although no unequivocal flow-limiting dissection was identified, the angiographic findings did not permit reliable differentiation between a subtle angiographically inapparent dissection and acute recoil. Because the flow impairment persisted, a 2.5 × 18 mm XIENCE (Abbott Vascular, Santa Clara, CA, USA) everolimus-eluting stent was implanted across the treated segment. Final angiography in two projections demonstrated visually satisfactory angiographic stent expansion, no visually apparent residual stenosis, and restoration of TIMI grade 3 flow.

The second lesion was 12 mm long and had 70–80% diameter stenosis. Predilatation with a 2.5 × 15 mm semi-compliant balloon resulted in approximately 10% residual stenosis. After inflation of a 2.5 × 20 mm MagicTouch sirolimus-coated balloon, repeat angiography demonstrated residual stenosis >30% accompanied by angiographic recoil. This finding persisted after intracoronary vasodilator administration and an approximately 5-min observation period, as confirmed in two angiographic projections. A 2.5 × 23 mm XIENCE everolimus-eluting stent was therefore implanted. Final angiography in two projections demonstrated visually satisfactory angiographic stent expansion, TIMI grade 3 flow, and no visually apparent residual stenosis. Following bailout stenting, neither patient developed postprocedural chest pain or new ischemic electrocardiographic changes, and no additional clinically apparent procedural complication was recorded.

### 3.6. Six- and Twelve-Month Clinical Outcomes

No TLF was recorded before either the 6- or 12-month landmark. Thirty patients were alive and under documented follow-up at both time points; one patient died from a non-cardiovascular cause at month 1. No clinically driven TLR, target-vessel myocardial infarction, or cardiovascular death was recorded within 12 months. No stroke or CABG occurred during follow-up. Continued use of the prescribed DAPT regimen was documented during follow-up, and no unplanned interruption was recorded. At month 20, one patient presented with chest pain at rest, without cardiac troponin elevation. Coronary angiography demonstrated approximately 70% restenosis at the previously DCB-treated diagonal-branch target lesion, with an angiographic appearance consistent with superimposed thrombus. Clinically driven TLR was performed with implantation of a 2.5 × 18 mm XIENCE everolimus-eluting stent, with restoration of TIMI grade 3 flow. The event occurred among the four patients with at least 20 months of record-based follow-up and was analyzed descriptively outside the prespecified 12-month endpoint window.

## 4. Discussion

Among 31 patients treated with an intended DCB-only strategy, 29 avoided stent implantation and two required bailout stenting. Final PCI was successful in all cases. No TLF was recorded within 12 months. The absence of recorded TLF at 12 months should be interpreted cautiously because of the small sample size, selected population, and absence of a comparator group. These observations show that DCB angioplasty was technically feasible in this selected cohort. They should not be read as an estimate of comparative efficacy because the study was small and had no DES control group.

Medina 0,0,1 lesions are uncommon, but the ostial location makes PCI difficult. A stent may miss part of the ostium or extend into a disease-free main vessel, and either problem can complicate future access to the side branch. Registry data also show clinically relevant rates of repeat revascularization and other adverse events after PCI [3,4]. DCB angioplasty avoids a permanent scaffold. Its limitation is equally clear: if balloon preparation leaves recoil, dissection, or reduced flow, drug delivery alone is not enough.

Vaquerizo et al. treated 49 patients with a second-generation paclitaxel-coated balloon. Seven patients (14.3%) required bailout stenting, mainly for recoil or dissection, and the 1-year TLR rate was 14.3% [7]. Direct comparison with our cohort is inappropriate because the studies differed in patient selection, sample size, device generation, lesion preparation, and follow-up. Kang et al. subsequently reported lower 2-year TLF with a DCB-based strategy than with DES in a propensity-adjusted observational analysis [8]. Akın et al. also reported high procedural success and one bailout stent in a 48-patient isolated side-branch cohort [11]. These studies place our results within a growing but still observational evidence base. None establishes DCB as superior to DES.

Randomized bifurcation trials address a different procedural setting. PEPCAD-BIF found less late lumen loss and angiographic restenosis with DCB than with plain balloon angioplasty after successful predilatation [12]. BEYOND also reported favorable angiographic findings after DCB treatment of the side branch [13]. In DCB-BIF, DCB treatment reduced major adverse cardiac events compared with a noncompliant balloon in compromised side branches after main-vessel stenting [14]. SPACIOUS reported acceptable side-branch results with both sirolimus- and paclitaxel-coated balloons in a hybrid strategy [15]. In these trials, the main vessel was usually stented. Their findings support the rationale for DCB use in bifurcations but cannot be transferred directly to an isolated DCB-only Medina 0,0,1 strategy.

Meta-analyses of DCB use in coronary bifurcations have reported heterogeneous angiographic and clinical findings. Some analyses suggest less side-branch late lumen loss, whereas others found no significant difference in late lumen loss but reported possible reductions in myocardial infarction or composite clinical events; anatomical settings, comparator strategies, and endpoint definitions varied substantially [16,17,18,19]. REC-CAGEFREE I did not show noninferiority of a DCB strategy with rescue stenting to planned DES implantation in unselected de novo noncomplex lesions at 2 years [20]. In the prespecified bifurcation analysis, DCB and DES had numerically similar clinical outcomes [21]. This difference matters. Current evidence does not support routine DCB-only PCI for all de novo lesions. It does justify further study of anatomies such as an isolated side-branch ostium, where avoiding metal addresses a specific procedural problem.

The difference between the prevalence of angiographically visible calcification and the frequency of non-compliant balloon use reflects the mild calcific burden in this cohort. In the retrospective review, an acceptable post-predilatation angiographic result was documented after semi-compliant balloon preparation in 7 of the 11 calcified lesions, whereas non-compliant balloons had been used in 4 lesions with relatively more pronounced, although still mild, angiographic calcification. All 11 lesions met the study-defined angiographic criteria for adequate preparation using conventional balloons; no additional calcium-modification technique had been used.

Lesion preparation was central in this series. A DCB has no scaffolding effect and cannot correct persistent recoil, a major dissection, or reduced coronary flow. Consensus documents recommend adequate predilatation, residual stenosis ≤30%, TIMI grade 3 flow, and no flow-limiting dissection before DCB-only completion [5,6,22,23]. Specialty balloons may assist lesion preparation in selected resistant lesions, but available evidence does not support their routine use before DCB treatment [24,25]. No definite flow-limiting dissection was identified in either bailout case. One patient had recoil with residual stenosis >30%; the other developed TIMI grade 2 flow after DCB. In both cases, the angiographic result—not adherence to a stentless plan—determined the need for a stent.

Bailout stenting was therefore treated as part of the procedural strategy. It counted as failure of DCB-only treatment but could still produce a successful final PCI result. DCB-only completion is reasonable only when lesion preparation produces adequate expansion, TIMI grade 3 flow, and no flow-limiting dissection. Persistent recoil, unacceptable residual stenosis, or impaired distal flow should lead to prompt stent implantation. This distinction should remain explicit when reporting DCB-only success and overall procedural success.

## 5. Study Limitations

This study should be interpreted in light of its retrospective, single-center design, modest sample size, and absence of a comparator group. Patients were selected for DCB treatment according to clinical and angiographic judgment; therefore, the findings primarily apply to carefully selected Medina 0,0,1 lesions and may not be generalizable to an all-comer population. The cohort predominantly comprised relatively short lesions in small-caliber side branches; therefore, the findings should not be extrapolated directly to longer, larger-caliber, or more complex ostial lesions. Two otherwise eligible patients without clinical follow-up were excluded, which may have introduced selection bias, although 12-month clinical status was available for all surviving patients in the analyzed cohort. Device selection, lesion preparation, and the decision to perform bailout stenting were operator-directed, reflecting real-world practice but limiting procedural standardization. Two DCB platforms with different antiproliferative drugs were used, and the small, markedly unbalanced platform groups precluded meaningful device-specific comparisons. Angiographic outcomes were assessed visually without an independent core laboratory, routine quantitative coronary angiography, or systematic intravascular imaging. Because postprocedural cardiac biomarkers were not routinely measured after elective procedures, clinically silent periprocedural myocardial infarction could not be assessed systematically. In addition, protocol-mandated angiographic follow-up was not performed; therefore, asymptomatic restenosis and late luminal changes could not be evaluated. One patient died from a non-cardiovascular cause at month 1, leaving 30 patients evaluable at 12 months. Follow-up beyond 12 months was not uniform, and the single 20-month TLR was therefore reported descriptively rather than as a time-to-event estimate. Despite these limitations, the observed procedural and clinical outcomes support the feasibility of an intended DCB-only strategy in appropriately selected patients. These findings should be considered hypothesis-generating and confirmed in larger prospective comparative studies.

## 6. Conclusions

An intended DCB-only strategy avoided stent implantation in 29 of 31 selected patients with isolated ostial side-branch Medina 0,0,1 lesions. Two mechanical failures—recoil with residual stenosis and deterioration of coronary flow—were managed with bailout stenting. No TLF was recorded within 12 months, although the cohort was small, selected, and lacked a comparator. These results support procedural feasibility. They do not establish superiority or clinical equivalence to DES. Prospective comparative studies are needed.

## Figures and Tables

**Table 1:** Baseline clinical, angiographic, and procedural characteristics.

Characteristic	Value
Clinical characteristics	
Age, years	65 (58–71)
Women	5/31 (16.1)
Diabetes mellitus	16/31 (51.6)
Hypertension	22/31 (71.0)
Hyperlipidemia	25/31 (80.6)
History of smoking	19/31 (61.3)
Previous percutaneous coronary intervention	16/31 (51.6)
Prior myocardial infarction	5/31 (16.1)
Stable clinical presentation	13/31 (41.9)
Acute coronary syndrome at presentation	17/31 (54.8)
Unstable angina	13/31 (41.9)
Non-ST-segment elevation myocardial infarction	2/31 (6.5)
ST-segment elevation myocardial infarction	2/31 (6.5)
Asymptomatic presentation	1/31 (3.2)
Left ventricular ejection fraction, %	56.5 (55–60); *n* = 30
Angiographic and lesion characteristics	
Target coronary territory	
Left anterior descending artery	18/31 (58.1)
Circumflex artery	11/31 (35.5)
Right coronary artery	2/31 (6.5)
Reference vessel diameter, mm	2.5 (2.25–2.5); *n* = 29
Lesion length, mm	18 (15–20); *n* = 28
Baseline diameter stenosis, %	80 (80–90)
Baseline TIMI flow grade 3	28/31 (90.3)
Baseline TIMI flow grade 2	2/31 (6.5)
Baseline TIMI flow grade 0	1/31 (3.2)
Mild angiographic calcification	11/31 (35.5)
Moderate or severe angiographic calcification	0/31 (0)
Thrombotic lesion	2/31 (6.5)
Procedural characteristics	
Sirolimus-coated balloon	28/31 (90.3)
Paclitaxel-coated balloon	3/31 (9.7)
First DCB diameter, mm	2.5 (2.25–2.5)
First DCB length, mm	20 (15–20)
Second DCB to complete longitudinal lesion coverage	3/31 (9.7)
Semi-compliant balloon preparation	27/31 (87.1)
Non-compliant balloon preparation	4/31 (12.9)
Residual stenosis after lesion preparation, %	10 (0–20)

Values are median (interquartile range) or *n*/N (%). Denominators are shown when data were not available for all 31 patients; denominator variation reflects missing documentation. Balloon-preparation categories were mutually exclusive. DCB, drug-coated balloon; TIMI, Thrombolysis in Myocardial Infarction.

**Table 2:** Immediate procedural and clinical outcomes.

Outcome	Result	Exact 95% CI, %
Immediate procedural outcomes		
DCB-only strategy success	29/31 (93.5)	78.6–99.2
Post-DCB angiographic success by visual estimation	29/31 (93.5)	78.6–99.2
Overall procedural success	31/31 (100.0)	88.8–100.0
Final TIMI 3 flow after DCB treatment	30/31 (96.8)	83.3–99.9
Type A dissection	2/31 (6.5)	0.8–21.4
Type B or type C–F dissection	0/31 (0)	0–11.2
Flow-limiting dissection	0/31 (0)	0–11.2
Bailout stenting	2/31 (6.5)	0.8–21.4
Bailout indication: deterioration to TIMI grade 2 flow	1/31 (3.2)	0.1–16.7
Bailout indication: persistent residual stenosis >30% with angiographic recoil	1/31 (3.2)	0.1–16.7
Emergency coronary artery bypass grafting	0/31 (0)	0–11.2
In-hospital death	0/31 (0)	0–11.2
Six-month clinical outcomes		
Target lesion failure	0 events	—
Clinically driven target lesion revascularization	0 events	—
Target-vessel myocardial infarction	0 events	—
Cardiovascular death	0 events	—
Twelve-month clinical outcomes		
Target lesion failure	0 events	—
Clinically driven target lesion revascularization	0 events	—
Target-vessel myocardial infarction	0 events	—
Cardiovascular death	0 events	—
Non-cardiovascular death	1/31 (3.2)	—
Stroke	0 events	—
Coronary artery bypass grafting	0 events	—
Beyond 12 months (descriptive)		
Clinically driven target lesion revascularization for target-lesion restenosis at 20 months	1 event	—

Exact confidence intervals were calculated for immediate procedural outcomes using the Clopper–Pearson method. Clinical outcomes are reported as event counts because one patient died from a competing non-cardiovascular cause before the 6- and 12-month landmarks; 30 patients were alive and under documented follow-up at both time points. Post-DCB angiographic success by visual estimation required residual stenosis ≤30%, final TIMI grade 3 flow, and no flow-limiting dissection or acute target-vessel occlusion. Overall procedural success permitted successful bailout stenting when an acceptable final PCI result was achieved without in-hospital cardiovascular death, urgent target lesion revascularization, or a major clinically apparent complication. The 20-month TLR for restenosis of the previously DCB-treated target lesion, with an angiographic appearance consistent with superimposed thrombus, was excluded from the prespecified 12-month endpoint. At the 20-month landmark, four patients remained under record-based follow-up; extended follow-up completeness is reported in Supplementary Table S1. DCB, drug-coated balloon; PCI, percutaneous coronary intervention; TIMI, Thrombolysis in Myocardial Infarction.

## Data Availability

The data that support the findings of this study are available from the corresponding author upon reasonable request and subject to institutional and ethical restrictions.

## Supplementary Materials

The following supporting information can be downloaded at: https://www.mdpi.com/article/10.3390/medicina62091620/s1. Supplementary Table S1: Completeness and duration of extended record-based clinical follow-up beyond 12 months.
