# Supplementary material for: Drug-Coated Balloon Angioplasty for Isolated Medina 0,0,1 Ostial Side-Branch Lesions: Procedural Outcomes, Bailout Stenting, and 12-Month Clinical Follow-Up
Source: Medicina (Kaunas). 2026 Aug 22;62(9):1620. doi: 10.3390/medicina62091620 (PMC13609164; doi:10.3390/medicina62091620)
Supplement: Supplementary file 1 [file medicina-62-01620-s001.zip › medicina-4491066-supplementary.pdf]

MDPI

medicina

Supplementary Materials

Drug-Coated Balloon Angioplasty for Isolated Medina 0,0,1 Ostial Side-Branch Lesions: Procedural Outcomes, Bailout Stenting, and 12-Month Clinical Follow-Up

Ebru Şahin *, Cansu Akdeniz, Begüm Sayın, Hakan Aksoy, Fehmi Kaçmaz and Ali Oto

Department of Cardiology, Memorial Ankara Hospital, Çankaya, 06520 Ankara, Türkiye

Supplementary Table S1. Completeness and duration of extended record-based clinical follow-up beyond 12 months.

Follow-up measure	Result
Analyzed cohort	31 patients
Twelve-month clinical status ascertained	31/31 (100%)
Patients alive with documented follow-up at 12 months	30/31 (96.8%)
Follow-up >12 months	19/31 (61.3%)
Follow-up ≥18 months	7/31 (22.6%)
Follow-up ≥20 months	4/31 (12.9%)
Follow-up ≥24 months	3/31 (9.7%)
Overall follow-up duration, months	13 (IQR, 12–17; range, 1–28)

Values are n/N (%) unless otherwise indicated. Twelve-month clinical status was ascertained for the entire analyzed cohort. One patient died from a non-cardiovascular cause at month 1 and was censored at death, leaving 30 patients alive with documented follow-up at 12 months. Follow-up duration was determined from the index procedure to the last documented clinical record, death, or the administrative data-closure date of 22 July 2026, whichever occurred first. Follow-up beyond 12 months was not uniform and was analyzed descriptively. At the 20-month landmark, four patients remained under record-based follow-up. IQR, interquartile range.
